# Incidence and Determinants of Piperacillin/Tazobactam-Associated Hypokalemia: A Retrospective Study

**DOI:** 10.3390/antibiotics11081138

**Published:** 2022-08-22

**Authors:** Heenam Seo, Eunyoung Kim

**Affiliations:** 1 Department of Pharmacy, Kangbuk Samsung Hospital, Seoul 03181, Korea; 2 Clinical Data Science, Evidence-Based Clinical Research, Regulatory Science Laboratory, Departments of Health Science, Clinical Pharmacy and Pharmacology, College of Pharmacy, Chung-Ang University, 84 Heukseok-ro, Dongjak-gu, Seoul 06974, Korea

**Keywords:** piperacillin/tazobactam, hypokalemia, incidence, risk factor, electrolyte disorder

## Abstract

Piperacillin/tazobactam (TZP) is a commonly used antibiotic for treating moderate-to-severe infections because of its broad-spectrum activity and recommendation as an alternative to carbapenem. TZP-associated severe hypokalemia has been consistently reported; however, related studies are very rare. This study aimed to evaluate the incidence and risk factors of TZP-associated hypokalemia (TAH). A retrospective cohort study was conducted on hospitalized adult patients who received TZP from 1 January 2015 to 31 December 2017 at a tertiary teaching hospital. Of the 713 patients, 13.9% had TAH. As a result of multivariate logistic regression analysis, older age (OR 1.03, 95% CI: 1.02–1.05, *p* < 0.001), female sex (OR 1.88, 95% CI: 1.18–3.00, *p* = 0.008), longer duration of TZP therapy (OR 1.08, 95% CI: 1.04–1.13, *p* < 0.001), and higher TZP daily dose (OR 1.10, 95% CI: 1.01–1.20, *p* = 0.049) were independently associated with TAH. In contrast, higher baseline serum potassium level (OR 0.13, 95% CI: 0.07–0.26, *p* < 0.001) was related to lower TAH. Furthermore, hypokalemia mostly occurred in the early days of TZP therapy (median onset time: 4 days). Thus, close monitoring of serum potassium levels, especially upon therapy initiation, is essential to prevent TAH.

## 1. Introduction

Hypokalemia is an electrolyte disorder commonly encountered in clinical practice. Mild hypokalemia is generally asymptomatic; however, severe hypokalemia might cause respiratory depression, paralytic ileus, and life-threatening cardiac arrhythmias [1,2,3,4]. Additionally, it has been reported that a low serum potassium level is associated with mortality and morbidity [5,6,7,8,9,10,11] in patients with cardiovascular disease, renal conditions, and others. Thus, it is important to identify or review the causes of hypokalemia to prevent or promptly and appropriately manage the disorder.

Piperacillin/tazobactam (TZP), a combination of semisynthetic ureidopenicillin and a β-lactamase inhibitor [12], is one of the most commonly used antibiotics for a variety of moderate-to-severe infections owing to its broad-spectrum activity. It is an alternative to carbapenems to prevent carbapenem resistance [13,14,15].

Pooled data from clinical trials indicated that TZP is generally safe and well tolerated [13]. However, TZP use has a variety of side effects [14]. Hypokalemia has been known as one of the rare electrolyte abnormalities of TZP. The reported incidence of TZP-associated hypokalemia (TAH) was 1.5% in patients with urinary tract infection [16] and less than 1% in patients with nosocomial pneumonia [14]. However, recent studies reported a notably high incidence of TAH. A randomized controlled trial comparing the efficacy and safety between fosfomycin injection and TZP in complicated urinary tract infections reported a 12.6% incidence of TAH [17]. Another retrospective study by Kuramoto et al. [18] assessing the incidence and risk factors of TAH reported a much higher incidence of 24.8%. In addition, serious TAH cases, mostly accompanied by arrhythmia and treatment discontinuation, have been continuously reported [19,20,21,22,23,24,25]. Nevertheless, the risk factors of TAH have not been well known because of the lack of related studies. However, Kuramoto et al. [18] study indicated older age was the only independent risk factor for TAH.

Considering the widespread use of TZP, it is essential to perform an in-depth analysis of TAH for the safe use of TZP. Therefore, we evaluated hypokalemia’s incidence and risk factors and assessed the time of TAH onset after starting TZP therapy.

## 2. Results

### 2.1. Demographic and Clinical Characteristics of Patients

**Figure 1:**
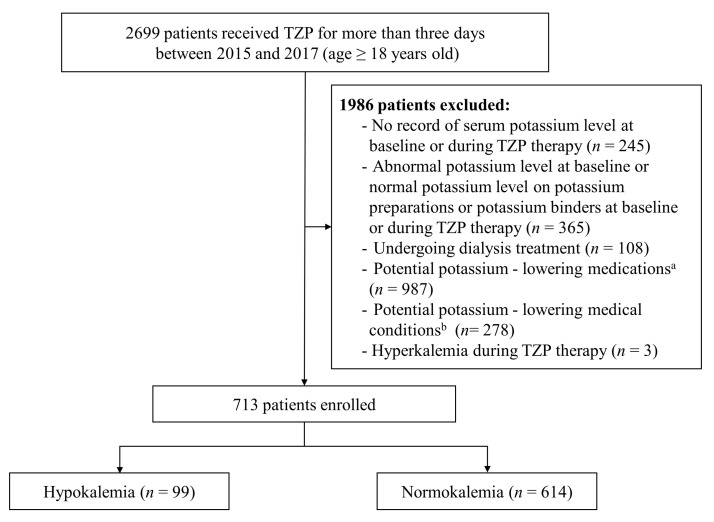
Patient flow diagram. TZP: piperacillin/tazobactam. ^a^ Diuretics (except for potassium-sparing diuretics alone), aminoglycosides, mineralocorticoids and glucocorticoids, amphotericin B, anticancer drugs, laxatives and enemas, β2 agonists, xanthines, and insulins were included; anticancer medications were cisplatin and other platinum drugs, cyclophosphamide, ifosfamide, cetuximab, panitumumab, temsirolimus, methotrexate, abiraterone, and eribulin. ^b^ Alkalosis, vomiting, diarrhea, tube drainage, hypomagnesemia, hypokalemic thyrotoxic periodic paralysis, primary hyperaldosteronism, Cushing’s syndrome, and renal tubular acidosis were included.

In total, 2699 adult patients were treated with TZP for more than three days between 1 January 2015 and 31 December 2017. Of these, 1986 patients were omitted based on the exclusion criteria. Finally, 713 subjects were included for analysis (Figure 1). The demographic and clinical characteristics of 713 patients at baseline are shown in Table 1. The median age of all patients was 67 years (interquartile range [IQR] 56–77 years), and 34.4% were female. Approximately 5.9% of patients stayed in the intensive care unit (ICU) while receiving TZP therapy. The baseline median serum potassium level was 4.2 mEq/L (IQR 3.9–4.5 mEq/L), and the baseline median serum creatinine level was 0.7 mEq/L (IQR 0.5–0.9 mEq/L). The median daily dose and duration of TZP therapy were 14.3 g (IQR 12.8–16.9 g) and 7 days (IQR 5–11 d), respectively. The most common indication for TZP therapy was pneumonia (37.3%). In addition, 314 (44.0%) of 713 patients were using at least one potential potassium-increasing medication, and the most common drug was non-steroidal anti-inflammatory drugs (NSAIDs) (26.5%).

### 2.2. Incidence and Severity of Hypokalemia

Of the 713 patients, hypokalemia occurred in 99 patients (13.9%); 9.3% of patients suffered from mild hypokalemia, while 4.6% had moderate-to-severe hypokalemia.

The comparison between the hypokalemic and the normokalemia groups is also presented in Table 1. The hypokalemia group included patients of significantly older age, females, with lower median baseline serum potassium level, longer median duration of TZP therapy, and a higher median TZP daily dose, compared to the normokalemia group.

### 2.3. Risk Factors of Hypokalemia

The results of univariate logistic regression revealed that older age, female sex, duration of TZP therapy, and TZP daily dose were associated with a higher risk of hypokalemia. In contrast, higher serum potassium levels at baseline were associated with a reduced risk of TAH (Table 2). There were no variables with VIF (>3); four statistically significant covariates from univariate analysis were included in the final multivariate logistic regression. Multivariate analysis also showed that, older age (odds ratio [OR] 1.03, 95% confidence interval [CI]: 1.02–1.05, *p* < 0.001), female sex (OR 1.88, 95% CI: 1.18–3.00, *p* = 0.008), longer duration of TZP therapy (OR 1.08, 95% CI: 1.04–1.13, *p* < 0.001), and higher TZP daily dose (OR 1.10, 95% CI: 1.01–1.20, *p* = 0.049) were positively associated with TAH, whereas higher baseline serum potassium levels (OR 0.13, 95% CI: 0.07–0.26, *p* < 0.001) was negatively associated with TAH (Table 2).

### 2.4. Other Outcomes

Our study indicated that hypokalemia occurred during the early days after the start of TZP therapy, with four days as the onset time and five days as the nadir time (Table 3). In addition, we identified that the decrease in serum potassium from baseline to nadir was 3.5 times greater in the hypokalemia group (0.7 mEq/L vs. 0.2 mEq/L, *p* < 0.001) with approximately three times higher in the proportion of patients with ≥0.5 mEq/L decrease in serum potassium after TZP therapy (82.8% vs. 28.8%, *p* < 0.001) (Table 4). 

## 3. Discussion

This is the first study to evaluate the incidence and associated risk factors of TAH in adult patients using data that excluded the medications and medical conditions influencing serum potassium levels with a large sample size. We found that hypokalemia was common in more than 10% of the patients and was more frequent in older and female patients, as well as in those with a longer duration of TZP therapy and higher TZP daily dose administration. In addition, we indicated that TAH occurred in the early days after TZP administration. These findings have important implications for the safe use of TZP.

The high frequency of hypokalemia in TZP treatment in our study is in line with the findings of previous studies [17,18]. The incidence of TAH in the present study was similar to the result of the study by Kaye et al. [17] (13.9% vs. 12.6%), whereas this was considerably lower than that by Kuramoto et al. [18] (13.9% vs. 24.8%). In terms of severity, the proportion of moderate-to-severe hypokalemia was much higher in our study compared to the findings by Kaye et al. [17] (4.5% vs. 1.3%). However, as expected, our result was lower than that of Kuramoto et al. [18] (4.5% vs. 6.4%). The differences in these results may be due to the differences in the inclusion cohorts between the studies.

In this study, females and older age were independent risk factors for developing hypokalemia in patients treated with TZP. In several studies, the female sex has been reported as a risk factor for hypokalemia [26,27,28,29]. Kleinfeld et al. [27] evaluated the relationship between the prevalence of hypokalemia and the age and sex of patients. They found that the incidence of hypokalemia was higher in females than in males, regardless of age. This might be due to differences in the body-mass composition [27]. Potassium content in the body is associated with muscle mass. Females, particularly elderly individuals, tend to have a lower body muscle mass than males or younger individuals, resulting in low total exchangeable potassium levels and a higher risk of developing hypokalemia [18,27,30]. A previous study suggested that a high body mass index (BMI) prevents hypokalemia occurrence [18]. However, in our study, body weight and BMI did not affect the TAH. It is a plausible result because weight and BMI do not reflect the body’s muscle mass. Older age was another risk factor for hypokalemia in the present study. This is consistent with the results obtained by Kuramoto et al. [18]. As mentioned above, the elderly may have lower body mass, and hypokalemia may occur more frequently [27]. In addition, they commonly present with malnutrition and chronic comorbidities with concomitant polypharmacy, such as insulin for diabetes mellitus, β agonists for pulmonary disease, and diuretics for several medical conditions [31], which affect the decrease in serum potassium level [27].

The occurrence of hypokalemia among patients treated with the drugs belonging to the penicillin class is typically known to be associated with high-dose therapy. This is because penicillin, including piperacillin in TZP, can act as non-reabsorbable anions that generate a transmembrane potential gradient in the cortical collecting tubules and augment potassium secretion, leading to hypokalemia [3,32]. This study also demonstrated that a higher dose therapy is a significant predictor of TAH.

In addition, prolonged TZP therapy was significantly associated with TAH in our study. A study comparing the safety of nafcillin and oxacillin also found that a longer duration of treatment was a risk factor for hypokalemia occurrence after adjusting for other confounding factors [33]. The underlying reasons for these results are not clear. However, the possibility of augmentation of potassium excretion due to drug accumulation by repeated administration may be considered.

Higher baseline serum potassium is essential in lowering hypokalemia development in TZP and other penicillin therapy [18,26,33]. The result of the present study corresponded well with the results of the previous studies.

Furthermore, we found that hypokalemia occurred early after the initiation of TZP therapy, and the median time to onset was four days. This result was generally consistent with those of previous studies. However, slight differences in the median onset time were observed: five days in the previous study with TZP [18], four days with oxacillin [33], and three days with nafcillin [33].

This study has some limitations. First, this is a retrospective study; therefore, individual researchers might have been subject to bias. Second, this is a single-center study; selection bias could affect its results, and therefore, for this reason, they might not be generalizable at the national or global level. Third, although we attempted to exclude as many factors affecting potassium levels as possible, excluding all possible contributors of hypokalemia except TZP was almost impossible. Patients treated with drugs that may increase serum potassium were not excluded. Therefore, caution is advised when generalizing the findings. Finally, we did not evaluate the complications of hypokalemia. Thus, further prospective studies are needed to determine more specific TAH-associated outcomes, including the clinical impact of hypokalemia.

## 4. Materials and Methods

### 4.1. Study Design and Population

A retrospective observational study was performed over three years (between 1 January 2015 and 31 December 2017) at Kangbuk Samsung Medical Center (Seoul, Korea), a tertiary-care teaching hospital with 800 beds.

Patients aged ≥18 years who received TZP for at least three days were included. Patients were excluded if they had no serum potassium level recorded at baseline or during TZP therapy; had abnormal serum potassium levels at baseline; maintained normal potassium levels with potassium preparations or potassium binders at baseline or during TZP therapy; and received dialysis and continuous renal replacement therapy. Kuramoto et al. [18] excluded patients on well-known hypokalemia-inducing drugs during TZP therapy to identify the incidence and risk factors of TAH. However, several medical conditions are also major causes of hypokalemia [1,3,34]. Thus, cases who received medications or had medical conditions that can contribute to hypokalemia development during TZP therapy were also excluded to specifically assess the impact of TZP on hypokalemia occurrence. The medications with the potential to decrease the serum potassium levels included diuretics (except for potassium-sparing diuretics alone), aminoglycosides, mineralocorticoids and glucocorticoids, amphotericin B, anticancer drugs, laxatives and enemas, β2 agonists, xanthines, and insulins [1,3,34]. Among anticancer drugs, the following medications reported in the literature for hypokalemia were excluded: cisplatin and other platinum drugs, cyclophosphamide, ifosfamide, cetuximab, panitumumab, temsirolimus, methotrexate, abiraterone, and eribulin [35]. Coexisting medical conditions that influence serum potassium levels were also considered. These included alkalosis, vomiting, diarrhea, tube drainage, hypomagnesemia, hypokalemic thyrotoxic periodic paralysis, primary hyperaldosteronism, Cushing’s syndrome, and renal tubular acidosis [1,3,34]. Finally, patients with hyperkalemia during TZP therapy were omitted.

This study was approved by the Institutional Review Boards of Kangbuk Samsung Medical Center (approval no. 2018-11-004). As this was a retrospective study, informed consent was not required. All clinical investigations were conducted per the guidelines of the 2008 Declaration of Helsinki.

### 4.2. Variables and Definitions

Data were extracted from electronic medical records. The collected data included patient demographic information (age, sex, weight, and height) and the status of ICU care during TZP treatment. It also included serum potassium, creatinine, magnesium, albumin, aspartate aminotransferase, and alanine aminotransferase levels; information related to TZP (daily dose, duration of therapy, and indication); and medications with the potential to increase the serum potassium levels, such as angiotensin-converting enzyme inhibitors (ACEIs), angiotensin II receptor blockers (ARBs), β-blockers, NSAIDs, digoxin, and sulfamethoxazole/trimethoprim [34,36]. BMI was calculated.

Hypokalemia and hyperkalemia were defined as serum potassium levels of <3.5 mEq/L and 5.5 > mEq/L, respectively. Alkalosis was defined as an arterial pH of >7.45. A serum magnesium level of <1.8 mg/dL was considered hypomagnesemia according to our institutional laboratory reference values. Patients included were classified into the normokalemia and hypokalemia groups based on potassium levels during TZP therapy. The hypokalemia group was further classified into mild (>3–3.4 mEq/L) and moderate-to-severe (≤3.0 mEq/L) groups. The baseline serum level was defined as the value recorded up to seven days before the start of TZP therapy. If multiple potassium levels were recorded before TZP therapy, the value obtained closest to the start of TZP therapy was considered the baseline.

### 4.3. Clinical Outcomes

The incidence, severity, and risk factors of TAH were evaluated. Additionally, clinical characteristics were assessed, including elapsed time to onset of hypokalemia and median difference of serum potassium before and after TZP therapy.

### 4.4. Statistical Analyses

Categorical variables were expressed as the frequency and proportion of variables and compared using the Pearson’s χ2 test or Fisher’s exact test. Continuous variables were described as the median and IQR and compared using the Mann–Whitney U test. To explore the risk factors, multivariate logistic regression was used. Variables with *p* < 0.05 from univariate analysis were included. However, variables with a variance inflation factor (VIF) exceeding 3 were excluded to avoid multicollinearity issues. Enter method was used. Statistical analyses were conducted using PASW Statistics 18.0 (IBM, Armonk, NY, USA). Statistical significance was set at *p* < 0.05 for two-tailed tests.

## 5. Conclusions

We found that the incidence of hypokalemia was considerably high in adult patients receiving TZP. Furthermore, we identified several factors affecting the onset of hypokalemia. In addition, we demonstrated that hypokalemia developed early under TZP treatment. Thus, close monitoring of serum potassium levels should be considered during TZP therapy, particularly at its beginning.

## Figures and Tables

**Table 1:** Demographic and clinical characteristics at baseline.

Variable	Total	Hypokalemia	Normokalemia	*p* Value
	*n* = 713	*n* = 99	*n* = 614	
Age (years)	67 (56–77)	74 (61.5–79.0)	66 (56–76)	<0.001
Female sex	245 (34.4)	46 (46.5)	199 (32.4)	0.006
ICU residence	42 (5.9)	5 (5.1)	37 (6.0)	0.702
Serum potassium (mEq/L)	4.2 (3.9–4.5)	3.9 (3.7–4.1)	4.2 (4.0–4.5)	<0.001
Serum creatinine (mEq/L)	0.7 (0.5–0.9)	0.7 (0.5–0.9)	0.7 (0.5–0.9)	0.707
Serum magnesium (mEq/L)	2.0 (1.9–2.2)	1.9 (1.8–2.1)	2.0 (1.9–2.2)	0.493
ALB (g/L)	3.2 (2.9–3.6)	3.1 (2.8–3.6)	3.2 (2.9–3.7)	0.675
AST (IU/L)	27 (18–44)	34 (18.8–53.5)	26 (17–43)	0.511
ALT (IU/L)	22 (18–38)	21 (11–38)	22 (15–38)	0.885
Duration of TZP therapy (days)	7 (5–11)	8 (5.5–13.0)	7 (5–11)	0.008
TZP daily dose (g)	14.3 (12.8–16.9)	15.8 (13.5–17.2)	13.5 (12.7–16.8)	0.019
BW (kg)	58 (50–66)	56 (50–64)	58.3 (50.2–66.1)	0.115
BMI (kg/m^2^)	21.9 (19.6–24.5)	22.2 (19.7–24.2)	21.9 (19.6–24.6)	0.824
Indication for TZP treatment				
Pneumonia	266 (37.3)	29 (29.3)	237 (38.6)	0.076
Intra–abdominal infection	197 (27.6)	35 (35.4)	162 (26.4)	0.064
Urinary tract infection	76 (10.7)	12 (12.1)	64 (10.4)	0.611
Neutropenic fever	42 (5.9)	5 (5.1)	37 (6.0)	0.702
Bacteremia/Sepsis	38 (5.3)	8 (8.1)	30 (4.9)	0.189
Skin and soft tissue infection	38 (5.3)	7 (7.1)	31 (5.1)	0.406
Others	81 (11.4)	8 (8.1)	73 (11.9)	0.268
Drugs that may increase serum potassium				
NSAIDs	189 (26.5)	32 (32.3)	157 (25.6)	0.158
ARBs	104 (14.6)	18 (18.2)	86 (14.0)	0.275
β blockers	28 (3.9)	2 (2.0)	26 (4.2)	0.293
Potassium-sparing diuretics	24 (3.4)	1 (1.0)	23 (3.8)	0.161
ACEIs	16 (2.2)	2 (2.0)	14 (2.3)	0.909
Sulfamethoxazole/trimethoprim	14 (2.0)	1 (1.0)	13 (2.1)	0.461
Digoxin	12 (1.7)	2 (2.0)	10 (1.6)	0.779

Data are presented *n* (%) or median (interquartile range); ICU: intensive care unit; ALB: albumin; AST: aspartate aminotransferase; ALT: alanine aminotransferase; TZP: piperacillin/tazobactam; BW: body weight; BMI: body mass index; NSAIDs: non-steroidal anti-inflammatory drugs; ARBs: angiotensin II receptor blockers; ACEIs: angiotensin-converting enzyme inhibitors.

**Table 2:** Logistic regression analysis of independent risk factors for TZP-associated hypokalemia.

Variable	Univariate Analysis		Multivariate Analysis ^a^	
	OR (95% CI)	*p* Value	OR (95% CI)	*p* Value
Age (year)	1.03 (1.01–1.05)	0.001	1.03 (1.02–1.05)	<0.001
Female sex	1.81 (1.18–2.78)	0.007	1.88 (1.18–3.00)	0.008
ICU residence	0.83 (0.32–2.16)	0.702		
Serum potassium (mEq/L)	0.15 (0.08–0.27)	<0.001	0.13 (0.07–0.26)	<0.001
Serum creatinine (mEq/L)	1.05 (0.64–1.71)	0.850		
Serum magnesium (mEq/L)	0.32 (0.02–7.05)	0.471		
ALB (g/L)	1.00 (0.61–1.65)	0.998		
AST (IU/L)	1.00 (0.99–1.00)	0.930		
ALT (IU/L)	1.00 (0.99–1.01)	0.943		
Duration of TZP therapy (days)	1.07 (1.03–1.11)	<0.001	1.08 (1.04–1.13)	<0.001
TZP daily dose (g)	1.10 (1.01–1.20)	0.024	1.10 (1.01–1.20)	0.049
Body weight (kg)	0.99 (0.97–1.0)	0.128		
BMI (kg/m^2^)	1.01 (0.95–1.07)	0.730		
Indication for TZP treatment				
Pneumonia	0.66 (0.42–1.05)	0.077		
Intra–abdominal infection	1.53 (0.97–2.39)	0.065		
Urinary tract infection	1.19 (0.62–2.29)	0.612		
Neutropenic fever	0.83 (0.32–2.16)	0.702		
Bacteremia/Sepsis	1.71 (0.76–3.85)	0.194		
Skin and soft tissue infection	1.43 (0.61–3.35)	0.408		
Others	0.65 (0.30–1.40)	0.271		
Drugs that may increase serum potassium				
NSAIDs	1.39 (0.88–2.20)	0.159		
ARBs	1.36 (0.78–2.39)	0.276		
β blockers	0.47 (0.11–2.0)	0.304		
Potassium-sparing diuretics	0.26 (0.04–1.96)	0.193		
ACEIs	0.89 (0.11–7.27)	0.909		
Sulfamethoxazole/trimethoprim	0.47 (0.06–3.65)	0.472		
Digoxin	1.25 (0.27–5.77)	0.779		

OR: odds ratio; CI: confidence interval: ICU: intensive care unit; ALB: albumin; AST: aspartate aminotransferase; ALT: alanine aminotransferase; TZP: piperacillin/tazobactam; BMI: body mass index; NSAIDs: non-steroidal anti-inflammatory drugs; ARBs: angiotensin II receptor blockers; ACEIs: angiotensin-converting enzyme inhibitors. ^a^ Values are adjusted for age, sex, baseline serum potassium level, duration of TZP therapy, and TZP daily dose.

**Table 3:** Clinical characteristics of patients with hypokalemia.

Characteristics	Value
Elapsed time to onset of hypokalemia (days)	4.0 (2.5–6.0)
Serum potassium at the onset time of hypokalemia (mEq/L)	3.3 (3.1–3.4)
Elapsed time to nadir of hypokalemia (days)	5.0 (3.0–7.0)
Serum potassium at the nadir time of hypokalemia (mEq/L)	3.2 (3.0–3.3)

Data are presented median (interquartile range).

**Table 4:** Comparison of clinical characteristics between hypokalemia group and normokalemia group.

Characteristics	Hypokalemia	Normokalemia	*p* Value
	*n* = 99	*n* = 614	
Difference between baseline and nadir serum potassium level	0.7 (0.5–1.0)	0.2 (0.0–0.5)	<0.001 ^a^
Patients with ≥0.5 mEq/L decrease in serum potassium level from baseline	82 (82.8)	177 (28.8)	<0.001 ^b^
Monitoring of serum potassium levels	4 (3–6)	3 (2–4)	<0.001 ^a^

Data are presented *n* (%) or median (interquartile range); ^a^ *p*-values from the Mann–Whitney U test; ^b^ *p* values from the χ2 test.

## Data Availability

The data presented in this study are available upon reasonable request from the corresponding author.

## References

[1] Weiner I.D., Wingo C.S. (1997). Hypokalemia––consequences, causes, and correction. J. Am. Soc. Nephrol..

[2] Kardalas E., Paschou S.A., Anagnostis P., Muscogiuri G., Siasos G., Vryonidou A. (2018). Hypokalemia: A clinical update. Endocr. Connect..

[3] Gennari F.J. (1998). Hypokalemia. N. Engl. J. Med..

[4] Hoskote S.S., Joshi S.R., Ghosh A.K. (2008). Disorders of potassium homeostasis: Pathophysiology and management. J. Assoc. Phys. India.

[5] Ahmed A., Zannad F., Love T.E., Tallaj J., Gheorghiade M., Ekundayo O.J., Pitt B. (2007). A propensity–matched study of the association of low serum potassium levels and mortality in chronic heart failure. Eur. Heart. J..

[6] Krogager M.L., Eggers-Kaas L., Aasbjerg K., Mortensen R.N., Køber L., Gislason G., Torp-Pedersen C., Søgaard P. (2015). Short–term mortality risk of serum potassium levels in acute heart failure following myocardial infarction. Eur. Heart J. Cardiovasc. Pharmacother..

[7] Luo J., Brunelli S.M., Jensen D.E., Yang A. (2016). Association between serum potassium and outcomes in patients with reduced kidney function. Clin. J. Am. Soc. Nephrol..

[8] Collins A.J., Pitt B., Reaven N., Funk S., McGaughey K., Wilson D., Bushinsky D.A. (2017). Association of serum potassium with all–cause mortality in patients with and without heart failure, chronic kidney disease, and/or diabetes. Am. J. Nephrol..

[9] Smith N.L., Lemaitre R.N., Heckbert S.R., Kaplan R.C., Tirschwell D.L., Longstreth W.T., Psaty B.M. (2003). Serum potassium and stroke risk among treated hypertensive adults. Am. J. Hypertens..

[10] Mattsson N., Kumarathurai P., Larsen B.S., Nielsen O.W., Sajadieh A. (2017). Mild hypokalemia and supraventricular ectopy increases the risk of stroke in community–dwelling wubjects. Stroke.

[11] Kieneker L.M., Eisenga M.F., Joosten M.M., de Boer R.A., Gansevoort R.T., Kootstra-Ros J.E., Navis G., Bakker S.J. (2017). Plasma potassium, diuretic use and risk of developing chronic kidney disease in a predominantly White population. PLoS ONE.

[12] Perry C.M., Markham A. (1999). Piperacillin/tazobactam: An updated review of its use in the treatment of bacterial infections. Drugs.

[13] Kuye O., Teal J., DeVries V.G., Morrow C.A., Tally F.P. (1993). Safety profile of piperacillin/tazobactam in phase I and III clinical studies. J. Antimicrob. Chemother..

[14] U.S. Food and Drug Administration Drug Approval Package: Zosyn (Piperacillin & Tazobactam). https://www.accessdata.fda.gov/drugsatfda_docs/nda/2005/050684_S045_050750_s012_ZosynTOC.cfm.

[15] Tamma P.D., Rodriguez-Bano J. (2017). The use of noncarbapenem β–lactams for the treatment of extended–spectrum β–lactamase infections. Clin. Infect. Dis..

[16] Kaye K.S., Bhowmick T., Metallidis S., Bleasdale S.C., Sagan O.S., Stus V., Vazquez J., Zaitsev V., Bidair M., Chorvat E. (2018). Effect of meropenem–vaborbactam vs piperacillin–tazobactam on clinical cure or improvement and microbial eradication in complicated urinary tract infection: The TANGO I randomized clinical trial. JAMA.

[17] Kaye K.S., Rice L.B., Dane A.L., Stus V., Sagan O., Fedosiuk E., Das A.F., Skarinsky D., Eckburg P.B., Ellis-Grosse E.J. (2019). Fosfomycin for injection (ZTI-01) versus piperacillin-tazobactam for the treatment of complicated urinary tract infection including acute pyelonephritis: ZEUS, a Phase 2/3 randomized trial. Clin. Infect. Dis..

[18] Kuramoto H., Masago S., Kashiwagi Y., Maeda M. (2019). Incidence and risk factors of hypokalemia in tazobactam/piperacillin–administered patients. Yakugaku Zasshi.

[19] Hussain S., Syed S., Baloch K. (2010). Electrolytes imbalance: A rare side effect of piperacillin/ tazobactam therapy. J. Coll. Phys. Surg. Pak..

[20] Zaki S.A., Lad V. (2011). Piperacillin–tazobactam–induced hypokalemia and metabolic alkalosis. Indian J. Pharmacol..

[21] Kutluturk F., Uzun S., Tasliyurt T., Sahin S., Barut S., Ozturk B., Yilmaz A. (2012). A rare complication of antibiotic (piperacillin/tazobactam) therapy: Resistant hypokalemia. J. Med. Cases.

[22] Kunder S.K., Chogtu B., Avinash A., Pathak A., Patil N., Adiga S. (2015). A case series of piperacillin–tazobactam induced hypokalemia in a tertiary care hospital in South India. Online J. Health Allied Sci..

[23] Kumar V., Khosla S., Stancu M. (2017). Torsade de Pointes induced by hypokalemia from imipenem and piperacillin. Case Rep. Cardiol..

[24] Pandya A.D., Gupta S., Malhotra S.D., Patel P. (2018). Piperacillin-tazobactam induced hypokalaemia. Int. J. Basic Clin. Pharmacol..

[25] Tai C.C., Chou R.Y., Guo J.Y., Chen H.P. (2020). Severe acute hypokalaemia associated with piperacillin/tazobactam in an HIV–infected patient under antiretroviral therapy with tenofovir alafenamide: Case report and literature review. Sex. Health.

[26] van der Heijden C., Duizer M.L., Fleuren H., Veldman B.A., Sprong T., Dofferhoff A., Kramers C. (2019). Intravenous flucloxacillin treatment is associated with a high incidence of hypokalaemia. Br. J. Clin. Pharmacol..

[27] Kleinfeld M., Borra S., Gavani S., Corcoran A. (1993). Hypokalemia: Are elderly females more vulnerable?. J. Natl. Med. Assoc..

[28] Paice B.J., Paterson K.R., Onyanga-Omara F., Donnelly T., Gray J.M., Lawson D.H. (1986). Record linkage study of hypokalaemia in hospitalized patients. Postgrad. Med. J..

[29] Fukui S., Otani N., Katoh H., Tsuzuki N., Ishihara S., Ohnuki A., Miyazawa T., Nawashiro H., Shima K. (2002). Female gender as a risk factor for hypokalemia and QT prolongation after subarachnoid hemorrhage. Neurology.

[30] Rundo J., Sagild U. (1955). Total and exchangeable potassium in humans. Nature.

[31] Gerçek A., Umuroğlu T., İnci F., Göğüs Y. (2003). The etiology and incidence of hypokalemia in intensive care unit. Marmara Med. J..

[32] Brunner F.P., Frick P.G. (1968). Hypokalaemia, metabolic alkalosis, and hypernatraemia due to “massive” sodium penicillin therapy. Br. Med. J..

[33] Viehman J.A., Oleksiuk L.M., Sheridan K.R., Byers K.E., He P., Falcione B.A., Shields R.K. (2016). Adverse events lead to drug discontinuation more commonly among patients who receive nafcillin than among those who receive oxacillin. Antimicrob. Agents Chemother..

[34] Gennari F.J. (2002). Disorders of potassium homeostasis. Hypokalemia and hyperkalemia. Crit. Care Clin..

[35] Liamis G., Filippatos T.D., Elisaf M.S. (2016). Electrolyte disorders associated with the use of anticancer drugs. Eur. J. Pharmacol..

[36] Perazella M.A. (2000). Drug-induced hyperkalemia: Old culprits and new offenders. Am. J. Med..

